# Self-identified African ancestry and pathomics-derived tumor-infiltrating lymphocyte scores in colon and rectal adenocarcinoma whole-slide images: an exploratory multicohort whole-slide image study

**DOI:** 10.3389/fonc.2026.1855135

**Published:** 2026-08-10

**Authors:** Yuwei Zhang, Xiaolu Cheng, Yunhan Liao, Seidu Adams, Dimitri F. Joseph, Ricardo E. Flores, Joseph F. LaComb, Julie M. Clark, Ellen Li, Agnieszka B. Bialkowska, Jie Yang, Qi Yu, David Irabor, Akinfemi Akingboye, Olorunseun O. Ogunwobi, Alexandra Guillaume, Brian Theisen, Rajarsi Gupta

**Affiliations:** 1 Department of Biomedical Informatics, Stony Brook University, Stony Brook, NY, United States; 2 Bioinformatics Shared Resource at Stony Brook Cancer Center, Stony Brook University, Stony Brook, NY, United States; 3 Biostatistics Shared Resource at Stony Brook Cancer Center, Stony Brook University, Stony Brook, NY, United States; 4 Department of Biochemistry and Molecular Biology, Michigan State University, East Lansing, MI, United States; 5 Department of Pharmacology and Toxicology, Michigan State University, East Lansing, MI, United States; 6 Department of Medicine, Division of Gastroenterology and Hepatology, Stony Brook University, Stony Brook, NY, United States; 7 Department of Surgery, Henry Ford Pancreatic Cancer Center, Henry Ford Health, Detroit, MI, United States; 8 Department of Family, Population and Preventive Medicine, Stony Brook University, Stony Brook, NY, United States; 9 Department of Medicine, Division of Gastroenterology and Hepatology, New York City (NYC) Health + Hospitals/Kings County Hospital Center, Brooklyn, NY, United States; 10 Department of Medicine, Division of Gastroenterology and Hepatology, The State University of New York (SUNY) Downstate Health Sciences University, Brooklyn, NY, United States; 11 University of Ibadan, Ibadan, Nigeria; 12 Department of Surgery, The Dudley Group National Health System Trust, Russells Hall Hospital, Dudley, United Kingdom; 13 College of Health and Life Sciences, Aston Medical School, Birmingham, United Kingdom; 14 Department of Pathology, Henry Ford Health, Detroit, Detroit, MI, United States; 15 Department of Pathology, Michigan State University, College of Human Medicine, East Lansing, MI, United States

**Keywords:** African ancestry, colorectal neoplasm, pathomics, RNA sequencing (RNA-seq), tumor infiltrating lymphocyte (TIL)

## Abstract

**Introduction:**

AI assisted computed higher tumor infiltrating lymphocyte% (TIL%) scores from hematoxylin and eosin (H&E) stained whole slide images (WSI) may serve as a useful biomarker for stratifying patients for treatment with immune checkpoint inhibitors.

**Methods:**

To test the hypothesis that self-identified African ancestry (AA) vs. European ancestry (EA) was associated with higher TIL% scores, WSI were assembled from three diverse cohorts of formalin-fixed paraffin embedded (FFPE) colon and rectal adenocarcinoma (COAD-READ) tumor tissues: 1.) 242 AA vs. EA WSI downloaded from The Cancer Genome Atlas (TCGA)-COAD-READ database; 2.) 97 independent US self-identified AA vs. EA WSI assembled from three US medical centers; 3.) 48 (of 51) Nigerian WSI assembled from a single Nigerian medical center. There were 33 self-identified AA WSI suitable for analysis in the TCGA cohort and 49 self-identified AA WSI in the US cohort. For the TCGA cohort, 241 whole transcriptome RNA-sequence data were generated from parallel frozen tumor samples collected alongside the FFPE samples. For the US cohort, 87 RNA-seq enriched by exome capture data were generated from the same FFPE blocks as WSI.

**Results:**

No difference in median TIL% scores was detected between the combined TCGA and US AA cohorts and the Nigerian cohort. Exploratory multiple regression analysis of the combined TCGA and US cohorts revealed that AA had a higher TIL% score (Estimated difference = 3.01%, 95% CI (0.54%, 5.49%), P-value = 0.0170), while controlling for MMR/MSI status and other covariates. However, this result needs to be interpreted with caution because of differences between the TCGA and US cohorts with respect to representation of self-identified AA. After adjustment for cohort in the model, the association of AA with a higher TIL% score was no longer significant (Estimated difference = 2.05%, 95% CI(-0.59%, 4.70%), P-value = 0.1277). Moderate correlations were detected in the TCGA and US cohorts analyzed separately between computed TIL% scores and CIBERSORTx estimates of lymphocyte and T-cell abundance. Moderate correlations were also detected between TIL% scores and CXCL10 and CCL5 gene expression values.

**Conclusions:**

These preliminary results underscore the need for expanding the representation of minority populations in publicly accessible datasets.

## Introduction

Tumor-infiltrating lymphocytes (TILs) are a critical component of the tumor immune microenvironment and have been suggested as important prognostic markers of colon and rectal adenocarcinoma (COAD–READ) survival and response to immune checkpoint inhibitors in addition to MMR/MSI status (1, 2). Although TILs could serve as a useful prognostic biomarker, they are not routinely reported in clinical pathology laboratories at US medical centers. This is because manual assessment of TILs is labor-intensive and subject to interobserver variability. Pathomics AI tools have been developed to map and quantify COAD–READ tumor cells and lymphocytes in hematoxylin and eosin (H&E) whole-slide images (WSIs) (3–7). Quantifying TIL% is a potentially cost-efficient alternative to descriptive and semiquantitative assessments of inflammation/TILs and/or utilizing immunohistochemistry (IHC) antibodies to label T- and B-cell markers (e.g., CD3, CD8, and CD20) (8).

Higher levels of TILs based on manual assessments have been reported for mismatch repair enzyme-deficient/microsatellite instability-high (MMR-d/MSI-H) COAD–READ (9). MMR/MSI status is routinely reported at US medical centers because it helps stratify COAD–READ patients for treatment with immune checkpoint inhibitors and as an initial screen for hereditary Lynch syndrome (10). Higher TILs based on manual assessments have been previously reported in US self-identified African ancestry vs. European ancestry COAD–READ samples (11).

An advanced version of a previously described pathomics AI workflow (3, 4) on 242 cases (33 African ancestry, 209 European ancestry) was recently applied to compute TIL% scores from WSIs of H&E-stained COAD–READ formalin-fixed paraffin-embedded (FFPE) tumor sections downloaded from the TCGA-COAD–READ platform. Because only 33 African ancestry COAD–READ WSIs were analyzable in TCGA, the same workflow was applied to an independent clinical cohort (*n* = 97; 49 African ancestry, 48 European ancestry) to improve representation (12). Given the genomic relatedness between Nigerians and self-identified US African ancestry, we also included Nigerian COAD WSIs to further assess TIL% patterns across ancestry-linked but environmentally distinct populations (13–17). The same pathomics AI workflow was therefore also applied to 51 archived Nigerian clinical COAD–READ clinical FFPE WSIs to compare TIL% scores between US self-identified African ancestry and TCGA self-identified African ancestry cohorts.

Parallel RNA-seq data were generated in both the TCGA and US cohorts but not the Nigerian cohort. Because of technical differences in generating and processing the RNA-seq data between the TCGA and US cohorts, correlation analysis between TIL% and CIBERSORTx estimates of lymphocytes and T cells and with *CXCL10* and *CCL5* gene expression values was conducted separately for each cohort.

## Materials and methods

### Ethics statement for TCGA, US, and Nigerian cohorts

This study was approved by the Stony Brook Institutional Review Board (sIRB2024-0020) with reliance forms reviewed by the Institutional Review Boards for Henry Ford Health and Michigan State University. No reliance form was required for New York City Health + Hospitals/Kings County Hospital Center since the research protocol included an honest broker who oversaw HIPAA compliance and was determined to be not human research by its Institutional Review Board (IRB1949860). The TCGA RNA-seq and WSI data were downloaded from the TCGA-COAD–READ dataset without a dbGAP data use agreement. The US COAD–READ de-identified archived clinical FFPE samples (12) were assembled with a waiver of consent after approval by each of the institutional IRBs (Kings County Hospital Center, Stony Brook University, Henry Ford Health Center). The Kings County Hospital IRB deemed the study to be not human research, the Stony Brook University IRB deemed the study exempt, and the Henry Ford Health IRB deemed the study to be expedited (because they had no policy for honest brokers). De-identified archived clinical Nigerian samples were collected with a waiver of consent at the University of Ibadan, and the study was approved by the University of Ibadan IRB (Irabor, NHREC/05/01/08a) and deemed not human research by the Michigan State University IRB (Ogunwobi, STUDY00009876). All the data/samples included in this study were adult (age of resection ≥ 18 years), treatment naive, and of self-identified African or European ancestry. Because this study was focused on sporadic COAD–READ, samples from patients with familial COAD–READ (e.g., FAP, Lynch syndrome) inflammatory bowel disease were excluded from the analysis. All the US cohort and Nigerian samples were from initial surgical resections of treatment-naive COAD–READ. There was no information on the TCGA cohort as to whether samples were from biopsy or surgical resection.

### De-identified clinical metadata linked to the TCGA, US, and Nigerian cohorts

The TCGA cohort coded de-identified clinical metadata were downloaded from the Genome Data Commons (GDC, https://portal.gdc.cancer.gov/) without a dbGAP data use agreement. The US cohort clinical metadata were collected at each of the four institutions using a common data dictionary by manual curation of electronic medical records supervised by pathologists at each institution. Honest brokers at Kings County Hospital and Stony Brook University linked the curated clinical metadata to the coded de-identified samples. At Henry Ford Hospital Center, Dr. Theisen supervised manual curation of the clinical metadata with the assistance of research coordinators and supervised linking of the clinical metadata to coded de-identified samples. De-identified abstracted pathology metadata were linked to the coded de-identified Nigerian samples at the University of Ibadan by Dr. Irabor. The independent categorical variables of interest were as follows: 1) self-identified ancestry (African vs. European), 2) MMR/MSI status (MMR-d/MSI-H vs. MMR-p/MSI-L + MSS), 3) categorical age of collection (<50 vs. 50–75 years, >75 years), 4) sex (male vs. female), 5) tumor location (right colon vs. left colon + rectum), 6) tumor stage (I + II vs. III + IV), and 7) tumor grade (G1 + G2 vs. G3 vs. mucinous). Self-identified ancestry was extracted from the TCGA metadata self-identified race (black vs. white). The US cohort self-identified ancestry was extracted from self-identified race (black vs. white) information in electronic medical records. Additionally, samples from self-identified Hispanics and/or Latinos were excluded because of possible Native American admixture. The Nigerian self-identified ancestry was 100% African. The rationale for categorizing tumor location as right colon vs. left colon + rectum was based on the observations that manually assessed TIL scores were higher in the right vs. left colon + rectum (18); MMR-d/MSI-H status was associated with right colon tumor location and with high TIL scores (9); and self-identified African ancestry was associated with right colon tumor location (19, 20). The rationale for categorizing age as <50, 50–75, and >75 years was based on age <50 years defining diagnosis of early-onset COAD–READ (21). Age 50–75 years corresponded to the United States Preventive Services Task Force recommendations for COAD–READ screening up to 2021, when it was lowered to include ages 45–49 years (22) and because uptake of the revised recommendations was reported to be low (23). The rationale for grouping tumor stage I + II vs. III + IV and for grouping was based on a previous G1 + G2 vs. G3 + mucinous adopted from a previous study linking manually assessed TIL scores to these two variables (24). To harmonize tumor grading vocabulary across all cohorts, well differentiated was termed G1, moderately differentiated was termed G2, and poorly differentiated was termed G3. Intermediate grade scoring (e.g., G1/G2) was categorized with the higher grade (e.g., G2). Because mucinous tumors were not uniformly assigned a grade across the three cohorts, any tumor categorized as mucinous was not assigned a tumor grade.

### Annotation of MMR/MSI status for the TCGA, US, and Nigerian cohorts

MSI status by PCR-capillary electrophoresis and MMR status by immunohistochemistry (IHC) are highly correlated, where MSI-H corresponds to MMR-deficient (MMR-d) and where combined microsatellite stable (MSS) and microsatellite instability-low (MSI-L) corresponds to MMR-proficient (MMR-p) (25). Because there was incomplete annotation of TCGA MSI status downloaded from the GDC, we completed annotation using previously reported PCR-capillary electrophoresis results (12, 26). For the few TCGA samples without PCR-capillary electrophoresis results, MSI-H vs. MSI-L + MSS status was determined using the Microsatellite Analysis for Normal Tumor InStability (MANTIS) scores in the TCGA-COAD–READ database (12, 27). The US cohort MMR/MSI status was determined primarily by MMR-IHC (MMR-d/MMR-p) as part of clinical care at the respective institutions. Because MMR/MSI status is not routinely determined in Nigerian medical centers, MMR-IHC for four proteins (MLH1, PMS2, MSH2, and MSH6) was performed on freshly cut FFPE sections at the Henry Ford Cancer Center Pathology Core and reviewed by a senior staff GI surgical pathologist (B. Theisen). Dr. Theisen classified 41 of 51 samples based on two antibody (PMS2 and MSH6) staining instead of four antibodies, because of poor control staining in several samples, as previously described (28).

### Pathomics AI TIL%, tumor %, and lymphocyte % scores of the TCGA, US, and Nigerian cohorts

The Gupta laboratory shared pathomics-derived TIL%, tumor %, and lymphocyte % scores for 242 TCGA-COAD–READ diagnostic DX1 H&E WSIs of self-identified African (black or African-American) and European (white) ancestry in the TCGA-COAD–READ dataset (https://rgdpl.bmi.stonybrook.edu/yproj/Tumor-TILs/). The Gupta laboratory (Y.Z. and R.G.) downloaded 411 TCGA-COAD and 166 TCGA-READ WSIs of diagnostic (DX1) H&E-stained FFPE tumor sections, and 347 WSIs were selected for further analysis after review by Dr. Gupta. The TCGA cohort was heterogeneous with respect to H&E staining and scanning protocols from multiple institutions. For example, TCGA WSIs were scanned at either 20× or 40× resolution. Of the 347 TCGA WSIs, 33 WSIs were collected from self-identified African ancestry and 209 WSIs were from self-identified European ancestry COAD–READ samples. All 242 TCGA WSIs included in this study were scanned at 40× resolution.

The Gupta lab workflow version consists of two separate convolutional neural network (CNN) models that detect 1) ≥2 TILs in 50 µm^2^ tiled patches (corresponding to 200 × 200 pixels at 40×) to generate a spatial lymphocyte/TIL probability map and 2) ≥1 colorectal adenocarcinoma cells in 87.5 μm^2^ tiled patches (corresponding to 350 × 350 pixels at 40×) to generate a spatial COAD–READ probability map for each H&E WSI. The first CNN model was carried out as previously described (4) and was trained on the TCGA pan-cancer WSI dataset, where self-identified European ancestry represented ~80% of the samples and self-identified African ancestry represented ~10% of the samples. Both CNN models in the Gupta workflow were trained on a private collection of 200 de-identified COAD–READ WSIs from the University of Pennsylvania (see Acknowledgements: D.O., A.R., and E.F.) without information on staining and scanning protocols or information on self-identified ancestry. The University of Pennsylvania, however, serves a heavily urban and diverse (including self-identified African ancestry) catchment area of Philadelphia (29), so it is likely that self-identified African ancestry WSIs were included in the training set.

In contrast to the TCGA cohort, there was much less variability with respect to H&E staining and scanning of the stained FFPE sections for the US and Nigerian cohorts. H&E staining was performed on freshly cut FFPE sections at either the Henry Ford Cancer Center Pathology Core (Henry Ford Hospital US samples and Nigerian samples) or the Stony Brook Cancer Center Pathology Core (Kings County Hospital US samples and Stony Brook University US samples). All the US and Nigerian cohort H&E-stained slides were scanned on a single instrument, an Aperio C2 (Leica Biosystems Imaging, Vista, CA, USA) at 40× resolution and stored as svs. files. All the US and Nigerian WSIs were analyzed using the same workflow used for the TCGA WSIs.

The Gupta workflow does not include stain normalization, such as Macenko or Vahadane algorithms (30, 31). The choice to omit explicit stain normalization was a deliberate methodological decision based on alternative strategies in the Gupta pipeline to mitigate systematic biases. First, the initial pipeline was trained on WSIs across 23 different cancer types from the TCGA database (4). Since TCGA comprises samples from hundreds of global institutions processed over decades, the model was exposed to extreme baseline color and scanning heterogeneity during training, forcing it to learn invariant morphological features rather than specific color domains. Second, instead of forcing images into a rigid normalized color space—which often introduces artificial artifacts or distorts true pathological structures—the training pipeline utilized extensive color data augmentation in the HSL (hue–saturation–lightness) space. This ensures the model remains resilient against the exact type of staining variations observed across our cohorts.

The number of tumor patches for the 242 TCGA WSIs ranged from 3,282 to 145,958. The number of tumor patches for the 97 US WSIs ranged from 7,614 to 137,705 tumor patches, and none were excluded from further statistical analysis. The number of tumor patches for the 51 Nigerian WSIs ranged from 198 to 89,226. Three Nigerian WSIs were excluded from further statistical analysis because of insufficient sampling, resulting in 48 Nigerian WSIs. Manual assessment of the TCGA WSIs was previously validated with manual qualitative assessment by Dr. Gupta. Manual assessment of the H&E-stained WSIs in the 97 US and 48 Nigerian cohort WSIs was conducted by a senior staff GI surgical pathologist (Dr. Brian Theisen) to the nearest 5% and 10% increments (24).

### Analysis of parallel RNA-sequence data for the TCGA TIL% and independent US FFPE TIL% cohorts

All but one TCGA-COAD–READ sample with a TIL% score had a matched RNA-seq dataset, generated from RNA extracted from parallel frozen tissue collected from the same patient as the FFPE section used for WSI analysis. RNA-seq data were downloaded as unstranded STAR raw counts using the TCGAbiolinks R/Bioconductor package (12, 32).

Eighty-seven of the 97 independent US COAD–READ FFPE samples with TIL% scores underwent RNA sequencing enriched by exome capture (33) at the van Andel Research Institute Genomics Core with equal representation of the following four groups: 1) African ancestry MMR-d/MSI-H, 2) African ancestry MMR-p/MSI-L + MSS, 3) European ancestry MMR-d/MSI-H, and 4) European ancestry MMR-p/MSI-L + MSS. US COAD–READ tumor RNA was extracted from parallel FFPE curls as previously described (12). Exome libraries were constructed with 100 ng of FFPE RNA (QC threshold DIV200% > 30%) and the SureSelect MAX RNA Library Preparation kit, followed by target capture with SureSelect Max Target Enrichment using Fast Hybridization (Agilent Technologies, Inc., Santa Clara, CA, USA). Exonic regions were captured using the SureSelect XT HS Human All Exon V8 panel, followed by an additional 12 cycles of PCR. Paired-end 75 bp sequencing was performed on an Element AVITI sequencer (Element Biosciences, San Diego, CA, USA) to an average raw depth of 45M paired reads per library. Base calling was performed on instruments using AVITI OS (v3.2.0). Output files were demultiplexed and converted to FASTQ format using Element Biosciences Bases2fastq v1.7.0. Quality metrics using FastQC (v.0.12.1) and summarized using MultiQC (version 1.33) revealed that duplicate reads did not exceed 50% (34, 35). Ribosomal RNA (rRNA) content was estimated using the bbduk.sh function from BBTools (version 39.37) with the human rRNA reference sequence obtained from NCBI (U13369.1), https://www.ncbi.nlm.nih.gov/nuccore/U13369.1?report=fasta. After removing rRNA reads from the raw FASTQ files, the remaining reads were aligned to the human reference genome (GRCh38.primary_assembly.genome.fa) using STAR (2.7.11b) (36) with gene annotations from GENCODE (gencode.v49.primary_assembly.annotation.gtf). The reference genome and annotation files were downloaded from the GENCODE website https://www.gencodegenes.org/human/. Aligned BAM files were sorted using SAMtools (1.22.1) (37), and alignment quality was assessed with QualiMap2 (38). Transcript abundance was quantified as transcripts per million (TPM) using Salmon (1.10.3) (39), while raw gene counts were generated with FeatureCounts (40). Raw counts were normalized using the trimmed mean of M-values (TMM) method implemented in the R package edgeR, termed nCPM (41). Because 15 Nigerian RNA samples that passed Genomics Core QC (DV200% > 30%) all had duplicate reads in the 90% range, further RNA sequencing enriched by exome capture of the Nigerian FFPE samples was not pursued. The link to the GitHub repository for RNA-seq processing is https://github.com/xcheng2/RNA-seq-processing. *CXCL1*0 and *CCL5* gene expression values were calculated as a log(*x* + 1) transformation: log_2_(nCPM + 1). Estimation of immune cell-type abundance (e.g., lymphocytes, T cells) from RNA-seq TPM data was conducted using CIBERSORTx (42).

### Statistical analysis

Descriptive statistics for computed TIL% scores in the TCGA, US, and Nigerian cohorts used the Wilcoxon rank-sum test for binary categorical variables and the Kruskal–Wallis test for polytomous categorical variables. The Wilcoxon rank-sum test followed by the Dwass, Steel, Critchlow-Fligner (DSCF) *post hoc* adjustment was used for pairwise comparison in TIL% between groups. A multiple linear regression model was used to explore the difference in pathomics-derived TIL% scores (the primary outcome) between ancestry or MMR/MSI status. Only the combined TCGA and US cohorts were included, and the Nigerian cohort was excluded in regression analyses requiring reliable MMR/MSI classification. Covariates with unadjusted *P*-values <0.2 in the univariate analyses (categorical age, tumor location, tumor stage, tumor grade) were included as adjustors in multivariable models testing differences in TIL% by ancestry or MMR/MSI status. Sex was not included in the regression models because of its unadjusted *P*-value >0.9 in the univariate analysis. To test whether the effect of ancestry on TIL% differed by MMR/MSI status, an ancestry * MMR/MSI interaction term was further examined in a second model, with the same covariates adjusted. Sensitivity analyses using a Box–Cox transformation on the primary outcome (pathomics-derived TIL%) were performed to assess the impact of non-perfect normality on the results. Model assumptions were examined through residual diagnosis. For linear regression models, an estimated difference >0 indicated a higher TIL% in African (or MMR-d/MSI-H) patients, while a difference <0 indicated a higher TIL% in European (or MMR-p/MSI-L + MSS) patients. All analyses were exploratory, and hence, all *P*-values reported were not adjusted for multiple testing. Statistical analysis was performed using SAS 9.4 (SAS Institute Inc., Cary, NC, USA), and the significance level was set at 0.05.

## Results

### Descriptive statistics for TIL% scores between the TCGA, independent US, and Nigerian FFPE cohorts

**Figure 1:**
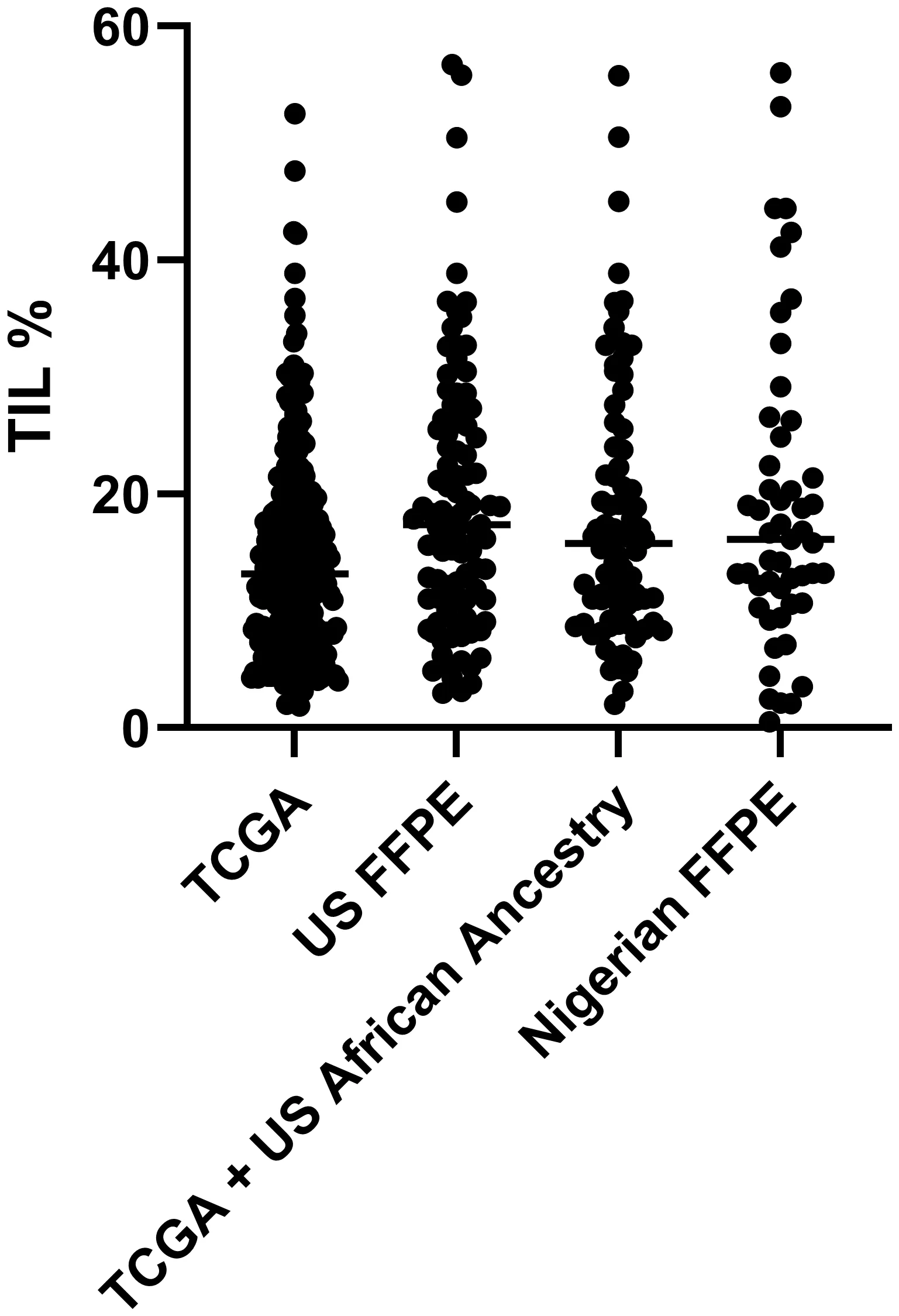
Scatter plot of pathomics-derived TIL% scores from TCGA (total), US (total), TCGA + US (combined) self-identified African ancestry, and Nigerian cohorts.

**Figure 2:**
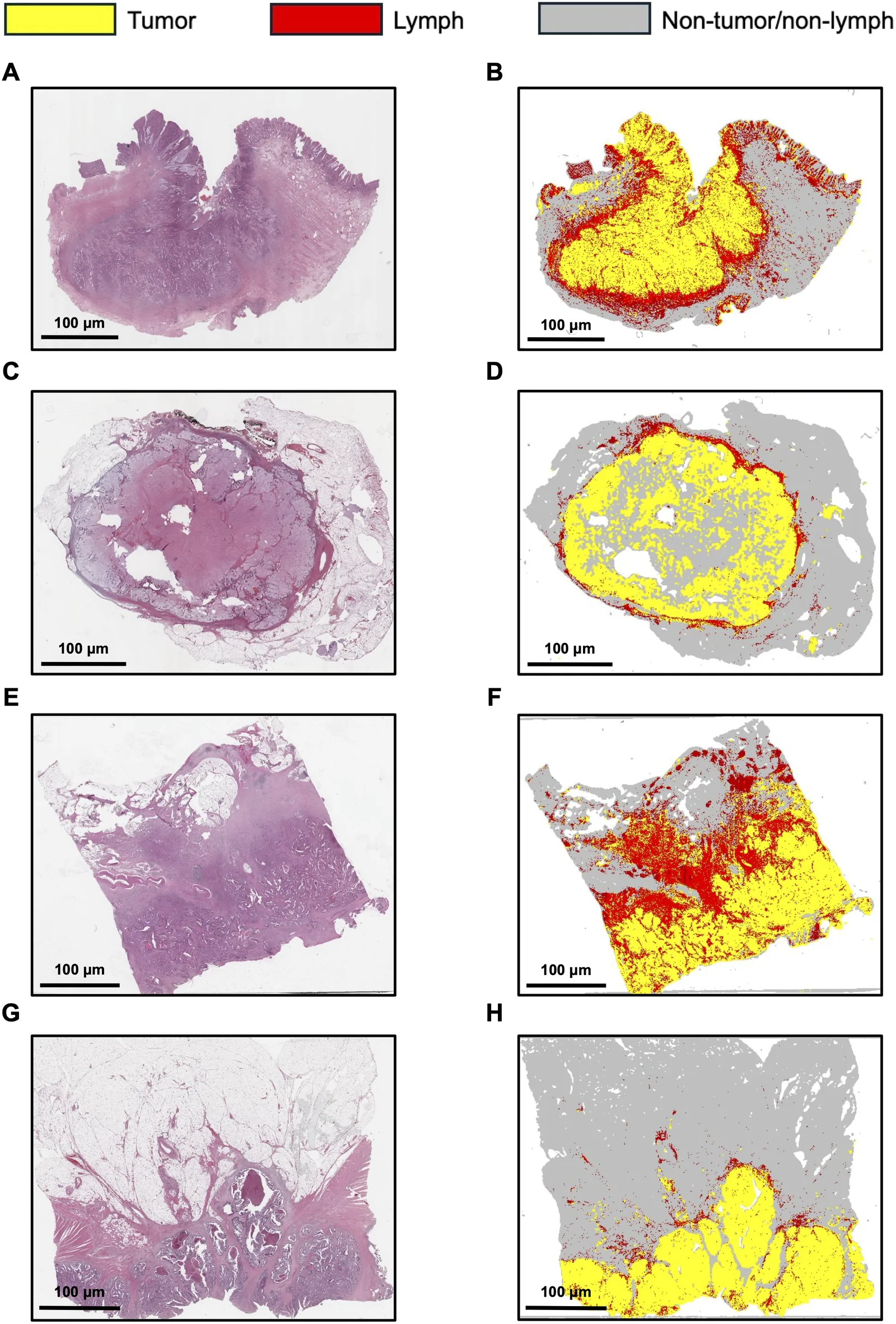
US self-identified African ancestry H&E-stained WSIs and corresponding tumor-TIL% maps. **(A)** MMR-d/TIL%-high H&E-stained WSI; **(B)** MMR-d/TIL%-high tumor-TIL map, TIL% = 27.6%; **(C)** MMR-d/TIL%-low H&E-stained WSI; **(D)** MMR-d/TIL%-low tumor-TIL map, TIL% = 7.7%; **(E)** MMR-p/TIL%-high H&E-stained WSI; **(F)** MMR-p/TIL-high tumor-TIL map, TIL% = 36.5%; **(G)** MMR-p/TIL%-low H&E-stained WSI; **(H)** MMR-p/TIL%-low tumor-TIL map, TIL% = 6.2%.

**Figure 3:**
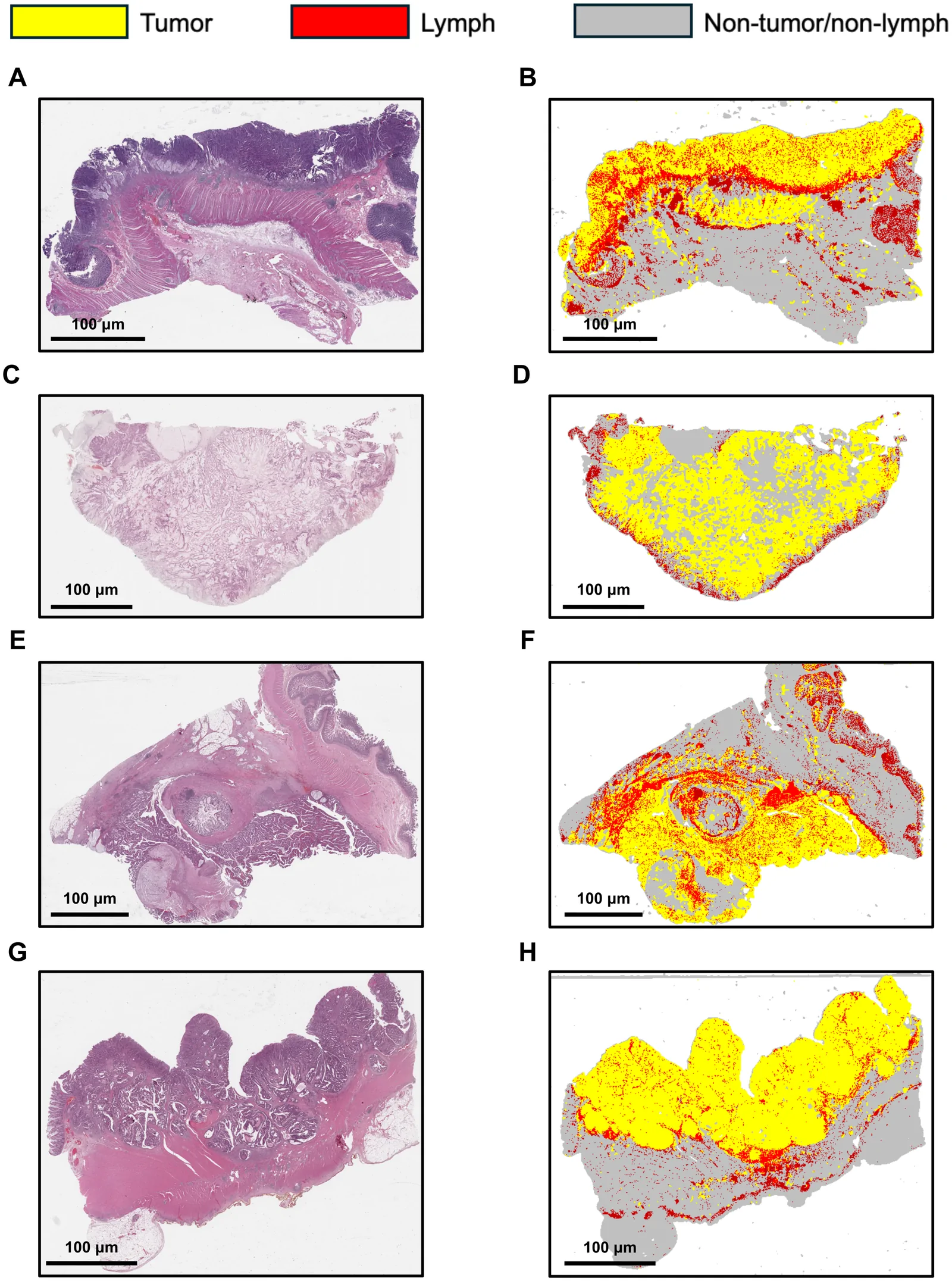
US self-identified European ancestry H&E-stained WSIs and corresponding tumor-TIL% maps. **(A)** MMR-d/TIL%-high H&E-stained WSI; **(B)** MMR-d/TIL%-high tumor-TIL map, TIL% = 23.3%; **(C)** MMR-d/TIL%-low H&E-stained WSI; **(D)** MMR-d/TIL%-low tumor-TIL map, TIL% = 3.0%; **(E)** MMR-p/TIL%-high H&E-stained WSI; **(F)** MMR-p/TIL-high tumor-TIL map, TIL% = 26.4%; **(G)** MMR-p/TIL%-low H&E-stained WSI; **(H)** MMR-p/TIL%-low tumor-TIL map, TIL% = 9.0%.

**Figure 4:**
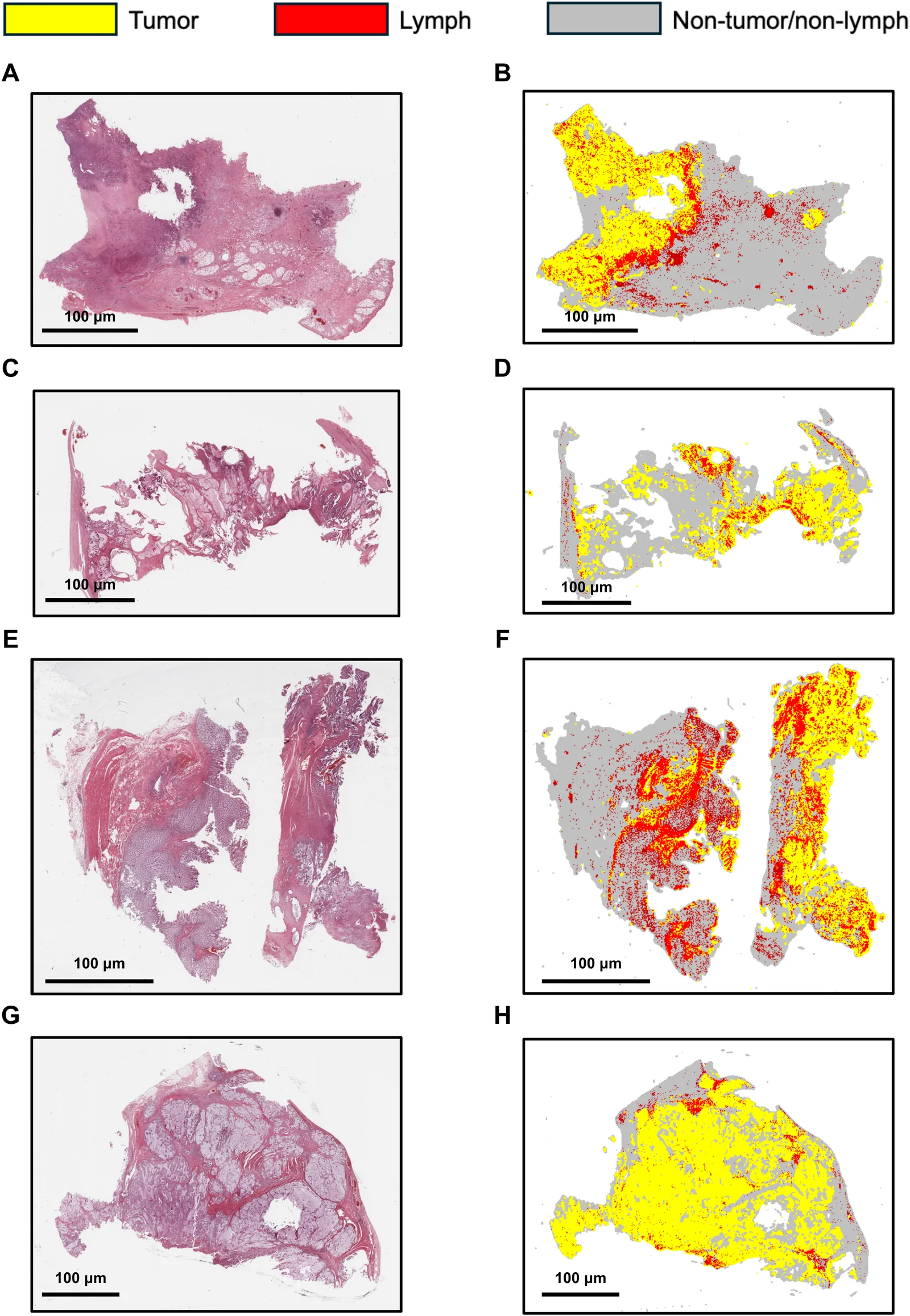
Nigerian H&E-stained WSIs and corresponding tumor-TIL% map. **(A)** MMR-d/TIL%-high H&E-stained WSI; **(B)** MMR-d/TIL%-high tumor-TIL map, TIL% = 19.2%; **(C)** MMR-d/TIL%-low H&E-stained WSI; **(D)** MMR-d/TIL%-low tumor-TIL map, TIL% = 13.2%; **(E)** US MMR-p/TIL%-high H&E-stained WSI; **(F)** MMR-p/TIL%-high tumor-TIL map, TIL% = 35.5%; **(G)** MMR-p/TIL%-low H&E-stained WSI; **(H)** MMR-p/TIL%-low tumor-TIL map, TIL% = 4.4%. Nigerian MMR-p samples shown were categorized based on all four antibodies in the panels.

A senior staff GI surgical pathologist manually assessed the IWTG TIL% scores to the nearest % increment (24) for the TCGA, US, and Nigerian cohorts to validate the Gupta pathomics pipeline. The Spearman correlation *ῤ* (95% CI) and unadjusted *P*-values between the pathomics-derived TIL% and the manually assessed IWTG TIL% scores were as follows: 1) *ῤ* = 0.8022, 95% CI (0.7506, 0.8441), unadjusted *P*-value <0.0001, *n* = 242 for the TCGA cohort; 2) *ῤ* = 0.7571, 95% CI (0.6467, 0.8293), unadjusted *P*-value <0.0001, *n* = 97 for the US cohort; and 3) *ῤ* = 0.6532, 95% CI (0.5476, 0.7383), unadjusted *P*-value <0.0001 for the Nigerian cohort. The high correlations with manually assessed TIL% values observed for each of the cohorts thus validate the Gupta pathomics pipeline. There was a significant difference in the pathomics-derived TIL% scores across the three cohorts (*P*-value = 0.0006, see **Supplementary Table 1**; **Figure 1**). Pairwise comparisons in TIL% scores between the three cohorts showed that the higher median TIL% ± IQR was only significant between US vs. TCGA patients (median ± IQR: 17.4% ± 14.9% vs. 13.2% ± 11.2%, unadjusted *P*-value = 0.0011). The differences for Nigerian vs. TCGA cohorts (16.4% ± 12.3% vs. 13.2% ± 11.2%, unadjusted *P*-value = 0.0621) and for US vs. Nigerian cohorts (17.4% ± 14.9% vs. 16.4% ± 12.3%, unadjusted *P*-value = 0.9644) did not reach significance. There was no significant difference in pairwise comparisons in TIL% scores between the combined TCGA and US self-identified African ancestry cohorts and the Nigerian cohort (15.7% ± 14.5% vs. 16.4% ± 12.3%, *P*-value = 0.5643, see **Supplementary Table 2**; **Figure 1**). Both the MMR-d/MSI-H and MMR-p/MSI-L + MSS groups had samples with TIL% scores above (TIL%-high) and below (TIL%-low) the median values for each cohort. Selected examples of matched H&E-stained WSIs with the corresponding tumor TIL% maps for MMR-d/TIL%-high, MMR-d/TIL%-low, MMR-p/TIL%-high, and MMR-p/TIL%-low are shown for the independent US African ancestry (see **Figure 2**), the US European ancestry (see **Figure 3**), and the Nigerian (see **Figure 4**) groups.

### Descriptive statistics of pathomics-derived TIL% scores in the combined TCGA and US FFPE cohorts for ancestry, MMR/MSI status, categorical age, sex, tumor location, tumor stage, and tumor grade

There were challenges to conducting MMR-IHC classification of the Nigerian samples. Sixty-two percent of the samples were re-embedded to section. Even after basing MMR-IHC classification on only two instead of all four antibodies because of poor control staining, 10 samples remained uncategorized. Only 2 of 38 Nigerian samples classified were MMR-d, which is much lower than previously reported Nigerian COAD–READ prevalence rates of MSI-H (14, 15). Consequently, the Nigerian cohort was excluded from further statistical analyses. While our primary focus was on ancestry and MMR/MSI status, we also included additional variables that have previously been reported to affect manually assessed TILs. When each variable was considered individually without controlling for other variables (see **Table 1**), the higher TIL% in African vs. European ancestry (15.7% ± 14.5% vs. 14.0% ± 12.0%, unadjusted *P*-value = 0.0719) did not reach significance. There was a significantly higher median TIL% score in the MMR-d/MSI-H vs. MMR-p/MSI-L + MSS groups (16.0% ± 14.4% vs. 13.5% ± 11.8%, unadjusted *P*-value = 0.0034). When ancestry was compared across MMR/MSI status (African ancestry MMR-d/MSI-H, African ancestry MMR-p/MSI-L + MSS, European ancestry MMR-d/MSI-H, European ancestry MMR-p/MSI-L + MSS), significant differences were also detected between the four groups (unadjusted *P*-value = 0.0146). Significant differences were detected between the three categorical age groups (<50, 50–75, and >75 years, unadjusted *P*-value = 0.0261). *Post hoc* pairwise comparisons between the three categorical age groups detected that the median TIL% score in the <50-year group was significantly lower than the >75-year group (unadjusted *P*-value = 0.0368). Higher TIL% was detected in the right vs. left + rectum tumor locations (15.6% ± 12.7% vs. 12.5% ± 12.0%, unadjusted *P*-value = 0.0072). Higher TIL% was detected in the early stage I + II group vs. the advanced stage III + IV group (15.4% ± 10.9% vs. 11.9% ± 10.7%, unadjusted *P*-value = 0.0019). TIL% differences between sex groups (male vs. female) and between tumor grade groups (G1 + G2 vs. G3 + mucinous) did not reach significance.

**Table 1:** Descriptive statistics of computed TIL% scores from combined TCGA and independent US cohort.

Variable	*N* missing	Level	*N*	Median TIL%	IQR	*P*-value*
Ancestry	0	African	82	15.7	14.5	0.0719
		European	257	14.0	12.0	
MMR/MSI status	6	MMR-d/MSI-H	79	16.0	14.4	0.0034
		MMR-p/MSI-L + MSS	254	13.5	11.8	
Ancestry by MMR/MSI status	6	African MMR-d/MSI-H	26	18.1	19.6	0.0146
		European MMR-d/MSI-H	53	15.3	12.7	
		African MMR-p/MSI-L + MSS	56	14.9	12.8	
		European MMR-p/MSI-L + MSS	198	13.2	11.5	
Categorical age	0	<50 years	40	11.6	8.9	0.0261
		50–75 years	215	14.0	12.0	
		>75 years	84	16.3	12.1	
Sex	0	Male	164	14.4	12.0	0.9130
		Female	175	14.1	12.1	
Tumor location	11	Right	181	15.6	12.7	0.0072
		Left + rectum	147	12.5	12.0	
Tumor stage	15	I + II	180	15.4	10.9	0.0019
		III + IV	144	11.9	10.7	
Tumor grade	13	G1 + G2	240	15.1	12.3	0.1940
		G3 + mucinous	86	13.7	11.7	

*P-values were based on the Wilcoxon rank-sum test for variables with two levels and based on Kruskal–Wallis for variables with >2 levels.

### Exploratory multiple linear regression models for testing whether pathomics-derived TIL% scores were significantly different between ancestry groups and between MMR/MSI status groups while controlling for covariates

A multiple linear regression model was then used to examine the difference in TIL% between ancestry (or MMR/MSI status), while controlling for other co-variables with unadjusted *P*-values <0.2 in univariate analyses: 1) categorical age, 2) tumor location, 3) tumor stage, and 4) tumor grade. In this initial model, self-identified African ancestry was associated with higher TIL% values (estimated difference = 3.01%, 95% CI: 0.54–5.49, *P*-value = 0.0170). Because ancestry was unevenly distributed between the TCGA and US cohorts, a cohort-adjusted sensitivity analysis was performed (see **Table 2B**). After adjustment for cohort, the association of self-identified African ancestry with a higher TIL% score was no longer significant (estimated difference = 2.05%, 95% CI: −0.59 to 4.70%, *P*-value = 0.1277). None of the other covariates, including MMR/MSI status and cohort, reached significance in either the initial or the cohort-adjusted sensitivity linear regression models.

**Table 2B:** Sensitivity analysis of multiple linear regression analysis (see **Table 2A**) with cohort adjusted.

Effect	Level	Estimated differences in TIL% (95% CI)	*P*-value*
Ancestry	African vs. European	2.05 (−0.59, 4.70)	0.1277
MMR/MSI status	MMR-d/MSI-H vs. MMR-p/MSI-L + MSS	1.89 (−0.94, 4.72)	0.1896
Categorical age	<50 vs. 50–75 years <50 vs. >75 years 50–75 vs. >75 years	0.60 (−2.78, 3.99) −1.41 (−5.34, 2.52) −2.02 (−4.65, 0.62)	0.3208
Tumor location	Right colon vs. left colon + rectum	0.24 (-2.17, 2.65)	0.8459
Tumor stage	I + II vs. III + IV	1.63 (−0.59, 3.85)	0.1489
Tumor grade	G1 + G2 vs. G3 + mucinous	1.53 (−1.02, 4.08)	0.2394
Cohort	US vs. TCGA	2.63 (−0.02, 5.28)	0.0521

This is a sensitivity analysis with cohort adjusted.

*P-values were from type 3 analysis based on a multiple linear regression model.

### Exploratory multiple linear regression models for testing whether pathomics-derived TIL% was significantly different between self-identified ancestry groups across MMR/MSI status while controlling for covariates

A second multivariable regression model was used for testing the effect of the first-order ancestry * MMR/MSI interaction term on TIL% scores while controlling for the same covariates (see **Table 3A**). Because the ancestry * MMR/MSI interaction term was not significantly associated with TIL% values, there is no clear evidence that the ancestry effect on TIL% differed by MMR/MSI status. Sensitivity analysis adjusting for cohort (see **Table 3B**) did not affect the conclusions from **Table 3A**.

**Table 3A:** Estimated differences and 95% CI in TIL% for ancestry across MMR/MSI status (first-order ancestry * MMR/MSI interaction) and additional covariates based on a multiple linear regression model.

Effect	Level	Estimated differences in TIL% (95% CI)	*P*-value*
African vs. European	MMR-d/MSI-H MMR-p/MSI-L + MSS	4.44 (−0.10, 8.97) 2.44 (−0.48, 5.35)	0.4624
Categorical age	<50 vs. 50–75 years <50 vs. >75 years 50–75 vs. >75 years	0.18 (−3.19, 3.55) −2.20 (−6.06, 1.66) −2.37 (−5.00, 0.25)	0.2005
Tumor location	Right colon vs. left colon + rectum	0.32 (−2.11, 2.75)	0.7987
Tumor stage	I + II vs. III + IV	1.66 (−0.58, 3.90)	0.1449
Tumor grade	G1 + G2 vs. G3 + mucinous	1.74 (−0.82, 4.29)	0.1817

*P-values were from type 3 analysis based on a multiple linear regression model.

**Table 3B:** Sensitivity analysis of multiple linear regression analysis (see **Table 3A**) with cohort adjusted.

Effect	Level	Estimated differences in TIL% (95% CI)	*P*-value*
African vs. European	MMR-d/MSI-H MMR-p/MSI-L + MSS	3.30 (−1.36, 7.97) 1.57 (−1.47, 4.61)	0.5230
Categorical age	<50 vs. 50–75 years <50 vs. >75 years 50–75 vs. >75 years	0.64 (−2.75, 4.03) −1.35 (−5.29, 2.59) −2.00 (−4.64, 0.64)	0.3287
Tumor location	Right colon vs. left colon + rectum	0.30 (−2.11, 2.72)	0.8047
Tumor stage	I + II vs. III + IV	1.70 (−0.53, 3.93)	0.1346
Tumor grade	G1 + G2 vs. G3 + mucinous	1.51 (−1.05, 4.06)	0.2460
Cohort	US vs. TCGA	2.58 (−0.08, 5.24)	0.0568

This is a sensitivity analysis with cohort adjusted.

*P-value was from type 3 analysis based on a multiple linear regression model.

Q–Q plots for the regression models used in **Tables 2A** and **3A** are shown in **Supplementary Figure 5** and residual vs. fitted plots are shown in **Supplementary Figure 6**. Residual diagnostic plots showed some mild deviation in the upper tail but did not show a clear non-linear pattern or obvious heteroscedasticity. The Q–Q plots showed some mild deviation in the upper tail but did not show a strong pattern, suggesting major violation of the linear regression assumptions. A few observations had relatively large studentized residuals, but most residuals were within a moderate range.

**Table 2A:** Estimated differences and 95% CI in TIL% for ancestry, MMR/MSI status, categorical age, tumor location, tumor stage, and tumor grade based on a multiple linear regression model.

Effect	Level	Estimated differences in TIL% (95% CI)	*P*-value*
Ancestry	African vs. European	3.01 (0.54, 5.49)	0.0170
MMR/MSI status	MMR-d/MSI-H vs. MMR-p/MSI-L + MSS	2.70 (−0.03, 5.42)	0.0542
Categorical age	<50 vs. 50–75 years <50 vs. >75 years 50–75 vs. >75 years	0.12 (−3.24, 3.49) −2.28 (−6.13, 1.57) −2.41 (−5.03, 0.21)	0.1890
Tumor location	Right colon vs. left colon + rectum	0.24 (−2.17, 2.65)	0.8462
Tumor stage	I + II vs. III + IV	1.58 (−0.65, 3.81)	0.1632
Tumor grade	G1 + G2 vs. G3 + mucinous	1.76 (−0.79, 4.31)	0.1745

*P-values were from type 3 analysis based on a multiple linear regression model.

Sensitivity analyses using multiple linear regression models with a Box–Cox transformation of pathomics-derived TIL% (*λ* = 0.2) assessed the impact of non-normality on the analysis results (see **Supplementary Tables 3, S4**). These sensitivity analyses using Box–Cox-transformed outcome produced similar conclusions to the original analyses that self-identified African ancestry was significantly associated with higher Box–Cox-transformed TIL% with an estimated difference of 0.285, 95% CI (0.016, 0.555), *P*-value = 0.0382. Similarly, the ancestry * MMR/MSI interaction term was not significantly associated with TIL% values. The only difference between the sensitivity Box–Cox-transformed analyses and the original untransformed analyses is that that the G1 + G2 tumor grade group was found to be positively associated with a higher Box–Cox-transformed TIL% score in both models, where ancestry and MMR/MSI status were considered separate fixed effect variables [estimated difference = 0.281, 95% CI (0.003, 0.559), *P*-value = 0.0476], and the second ancestry * MMR/MSI interaction model [estimated difference = 0.279, 95% CI (0.001, 0.558), *P*-value = 0.0495].

### Correlation of pathomics-derived TIL% scores from H&E-stained WSIs and CIBERSORTx lymphocyte and T-cell abundance from RNA-seq data calculated separately for the TCGA and the independent US cohorts

**Figure 5:**
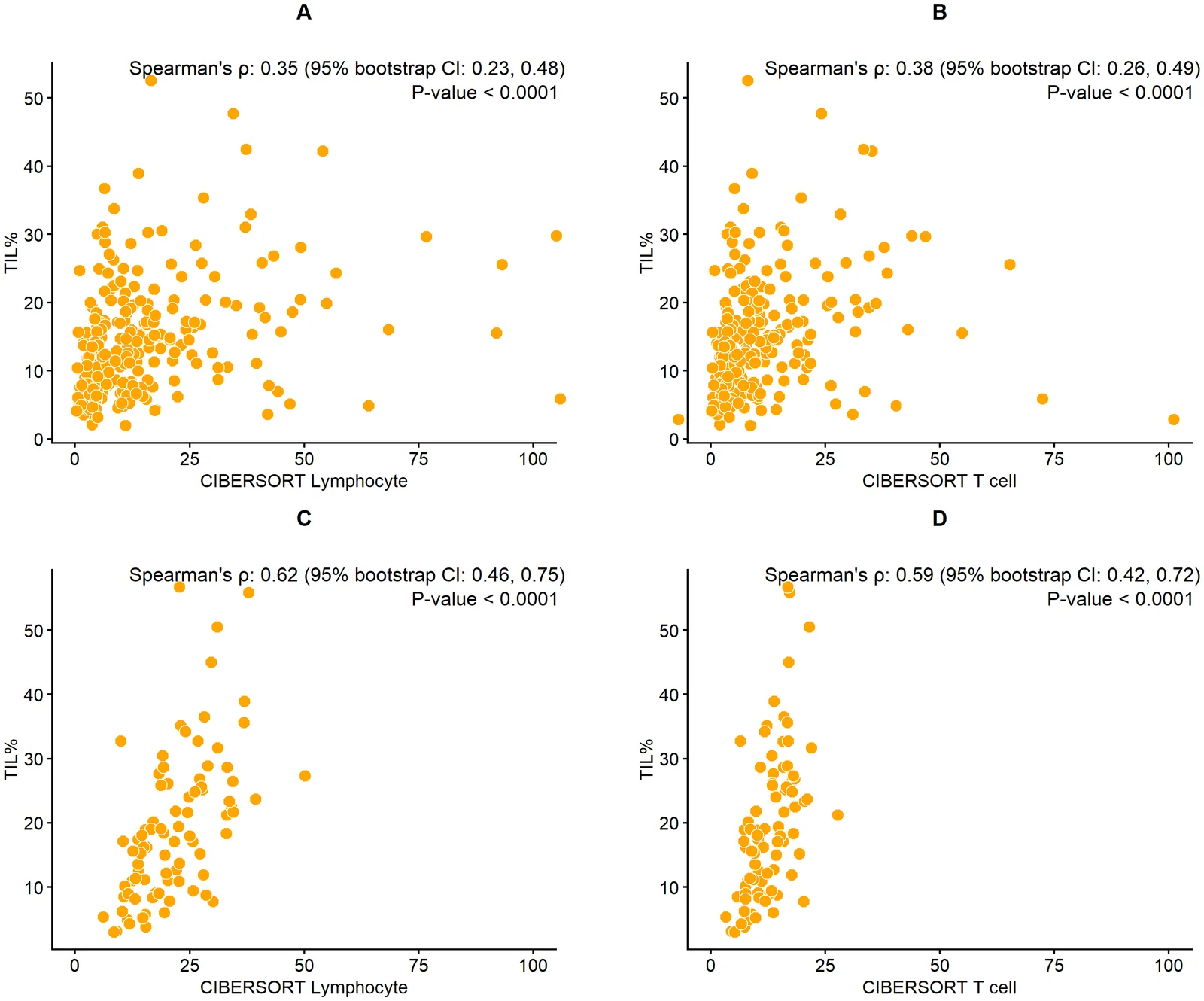
Scatter plots of Spearman correlation *ῤ* (95% CI) between pathomics-derived TIL% scores and RNA-seq-derived lymphocyte and T-cell abundance for the TCGA cohort and the US cohort. **(A)** TCGA TIL% score vs. CIBERSORTx lymphocytes; **(B)** TCGA TIL% score vs. CIBERSORTx T cells; **(C)** US TIL% score vs. CIBERSORTx lymphocytes; **(D)** US TIL% score vs. CIBERSORTx T cells. The TCGA cohort had 241 matched RNA-seq data out of a total of 242 WSIs. The US cohort had 87 matched RNA-seq data out of a total of 97 WSIs. The Spearman correlation *ῤ* (95% CI) and unadjusted *P*-values are shown on each panel.

The pathomics-derived TIL% scores should correlate with CIBERSORTx measurements of lymphocyte and T-cell abundance in parallel bulk RNA-seq datasets. As shown in **Figure 5**, both the TCGA and US cohort pathomics-derived TIL% scores correlated moderately with CIBERSORTx lymphocyte and T-cell abundance estimates, with Spearman *ῤ* correlations ranging from 0.35 to 0.62 and an unadjusted *P*-value threshold of 0.05. Given that all the unadjusted *P*-values were <0.0001, these correlations remained significant after reducing the Bonferroni threshold for significance to <0.0125 for four multiple comparisons.

### Correlation of pathomics-derived TIL% scores from H&E-stained WSIs and *CXCL10* and *CCL5* gene expression values from RNA-seq data calculated separately for the TCGA and the independent US cohort

**Figure 6:**
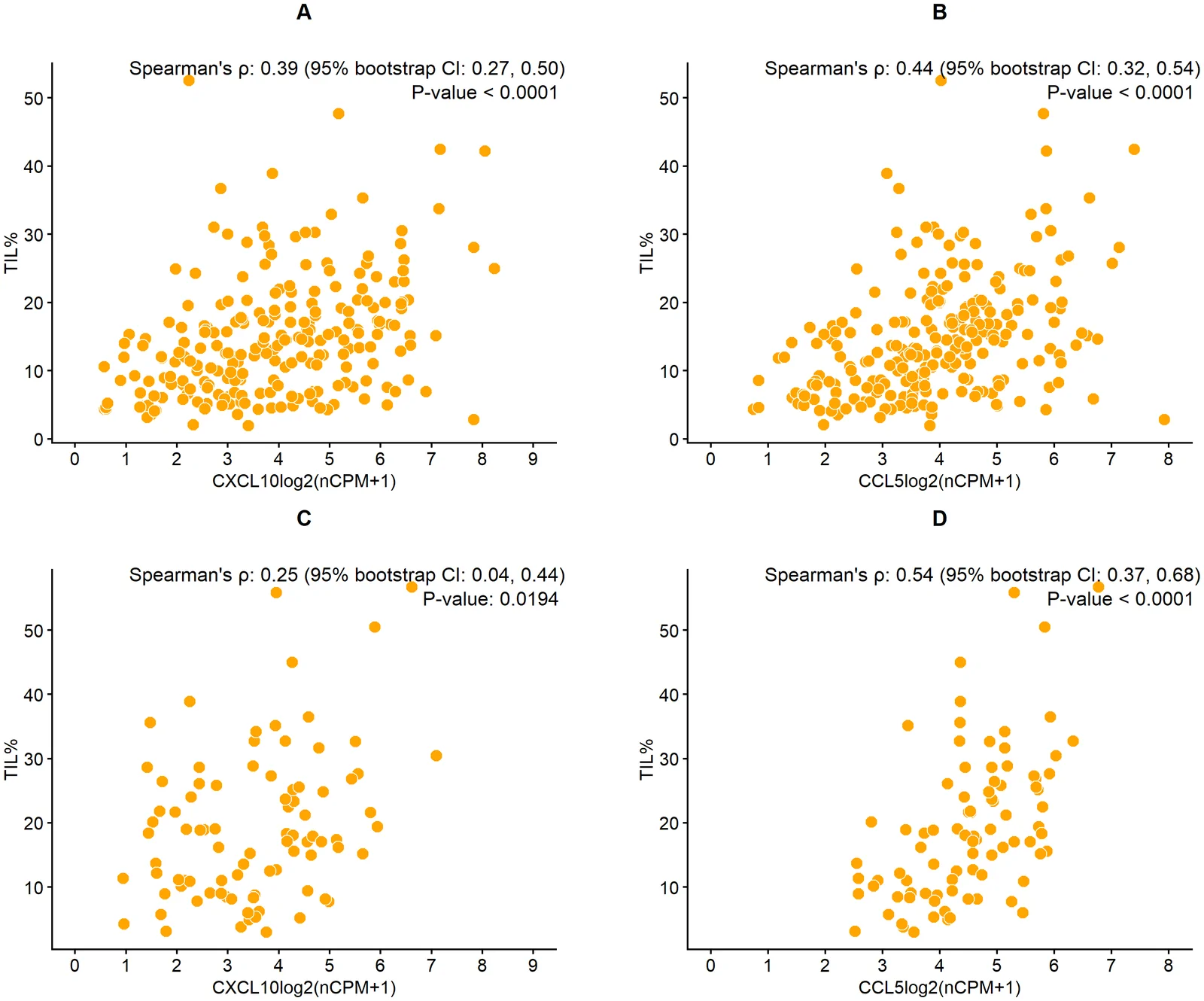
Scatter plots of Spearman correlation *ῤ* (95% CI) between pathomics-derived TIL% scores and RNA-seq-derived *CXCL10* and *CCL5* gene expression values [log_2_(nCPM + 1)] for the TCGA cohort and the US cohort. **(A)** TCGA TIL% score vs. CXCL10 log_2_(nCPM + 1); **(B)** TCGA TIL% score vs. CCL5 log_2_(nCPM + 1); **(C)** US TIL% score vs. CXCL10 log_2_(nCPM + 1); **(D)** US TIL% scores vs. CCL5 log_2_(nCPM + 1). The TCGA cohort had 241 matched RNA-seq data points out of a total of 242 WSIs. The US cohort had 87 matched RNA-seq data points out of a total of 97 WSIs. The Spearman correlation *ῤ* (95% CI) and unadjusted *P*-values are shown on each panel.

Because CXCL10 and CCL5 play key roles in antitumor immunity as T-cell attractants, their gene expression values should also correlate with the pathomics AI-computed TIL% scores (43, 44). As shown in **Figure 6**, both the TCGA and US cohort-computed TIL% scores correlated moderately with *CXCL10* log_2_(nCPM + 1) and *CCL5* log_2_(nCPM + 1) values, with Spearman *ῤ* correlations ranging from 0.25 to 0.54 and an unadjusted *P*-value threshold of <0.05. After reducing the Bonferroni threshold to <0.0125 for four multiple comparisons, the correlation between *CXCL10* log_2_(nCPM + 1) values and pathomics-derived TIL% scores (Spearman *ῤ* = 0.25, 95% CI: 0.04–0.44, unadjusted *P*-value = 0.0194) no longer reached significance.

## Discussion

The primary motivation for this study was to advance pathomics AI validation across ancestrally diverse populations by expanding representation of self-identified African ancestry in both US clinical and African cohorts. This helps us to establish whether TIL% scoring algorithms generalize beyond the predominantly European ancestry datasets on which they were developed. The second motivation was that TIL assessment, when combined with MMR/MSI status, may improve stratification of patients for immune checkpoint inhibitor therapy (45). With the addition of an independent US (FFPE) cohort, representation of self-identified US African ancestry samples with parallel H&E WSIs and RNA-sequencing datasets was increased from 33 in the TCGA to 82. Application of pathomics AI workflows to automate computation of TIL scores could potentially make it feasible to report this result in clinical pathology reports. For these workflows to be clinically useful for patient stratification, analyses need to be expanded across larger and more diverse cohorts. Currently, available WSI datasets are overwhelmingly European ancestry predominant.

While multiple AI workflows have been reported, not all workflows are suitable for evaluation of TILs in COAD–READ tumor tissues. For this study, the most recent pathomics AI workflow developed by the Gupta research group was chosen over other workflows because the senior staff surgical pathologist on this research team could access and review individual TIL scores and images from the TCGA cohort. As described in the *Methods* section, the Gupta workflow has been previously trained on the TCGA pan-cancer cohort that is heterogeneous with respect to staining and scanning protocols. In contrast to the TCGA, where the uploaded WSIs from diagnostic FFPE sections (DX1) were stained and scanned at multiple different institutions without a standardized protocol, the US and Nigerian cohort WSIs were generated using standardized H&E staining protocols at only two major medical centers (Henry Ford Health and Stony Brook Cancer Centers) and a single slide scanning instrument at a single location. While AI-assisted pathomics scoring of TILs has been performed thus far on self-identified European ancestry predominant cohorts, these cohorts were not exclusive of self-identified African ancestry populations. Although there is no information on the University of Penn WSIs that were used as a training set for the Gupta workflow, the patient demographics for this institution’s catchment area (29) make it likely that self-identified African ancestry COAD–READ WSIs were also included in this cohort.

The primary objective of our exploratory multivariable linear regression models was to test the hypothesis that self-identified African ancestry and/or MMR-d/MSI-H status were associated with higher pathomics-derived TIL% scores while controlling for other variables that have also been previously linked to manually assessed TIL scores. Only the TCGA and US combined cohorts were included in the multivariable models, because the MMR-IHC results for the Nigerian cohort were deemed unreliable. Thus far, one previous study conducted at a single center reported higher manually assessed TIL scores in self-identified African (*n* = 52) vs. European ancestry (*n* = 159) COAD–READ samples (11). Increased manually assessed TILs have been previously associated with MMR-d/MSI-H status (9). Tumor location, tumor stage, and tumor grade were included in our exploratory linear regression models because these variables have previously been reported to be associated with manually assessed TIL scores (24). While biological sex was included in the univariate analyses, it was not included in the multivariable regression models because the unadjusted *P*-values were high (>0.9). Because of concerns regarding the reliability of the MMR-IHC results in this Nigerian cohort, it was not included in further statistical analysis on the effect of self-identified ancestry and MMR/MSI status on the pathomics-computed TIL% scores.

Higher pathomics-derived TIL% values in the MMR-d/MSI-H group reached significance, but higher TIL% values in self-identified African ancestry groups did not reach significance, when each variable was analyzed separately, based on Wilcoxon rank-sum test *P*-values. Because the cohort was collinear with ancestry and ancestry was a primary variable of interest, cohort was not included in the initial linear regression model. While this initial model confirmed a previous study (11) that reported higher manually assessed TILs in self-identified African ancestry samples, our results should be interpreted with caution. This is because sensitivity analysis with adjustment for cohort rendered the association with self-identified ancestry no longer significant. These findings suggest that the initially observed self-identified ancestry association may have been influenced by cohort-related differences and should be interpreted cautiously. Larger studies with more balanced representation across ancestry groups will be needed to confirm these initial observations. Other covariates such as MMR/MSI status, tumor location, tumor stage, and tumor grade have been previously linked to manually assessed TILs and could also potentially contribute to the “unmasking” of self-identified African ancestry in the initial linear regression model. No significant association between self-identified ancestry * MMR/MSI interaction term and TIL% values was found. Thus, there is no clear evidence that self-identified African ancestry affects TIL% values across MMR/MSI status. Sensitivity analyses performed with Box–Cox transformations of the TIL% values show that skewness of the primary outcome (pathomics-derived TIL% scores) did not change these conclusions.

Correlation of the computed TIL% values with RNA sequence-derived estimates of lymphocyte and T-cell abundance in both the TCGA and US FFPE cohorts serves to further validate the pathomics AI workflow that was applied in this study. Furthermore, correlation of the computed TIL% scores with RNA-seq-derived *CXCL10* and *CCL5* RNA expression values provides further *in vivo* evidence of the roles played by these two chemokines in regulating antitumor immunity (43, 44). While this study increased the number of US African ancestry COAD–READ-derived WSIs and parallel RNA-seq data, the number of African ancestry samples is still limited. Having adequate representation of diverse populations is important for setting thresholds of computed TIL% scores as biomarkers to be used clinically in combination with MMR/MSI status (45). Another limitation is that the current version of the pathomics AI workflow does not further distinguish between different lymphocyte types. A previous immunohistochemical study reported no increased tumor infiltration of CD8-positive (T-cell marker) cells in MSS African ancestry COAD–READ FFPE sections but detected lower infiltration of granzyme B-positive cells compared with MSS European ancestry FFPE sections (46). Genomic-based estimates of continental African ancestry are a more objective method compared to self-identified ancestry. Self-identification of African ancestry in the US has been reported to average 73% genomic ancestry but can range from 30% to 100% (47, 48). Finally, this research team has delayed the collection of survival outcomes until the follow-up interval has reached at least 5 years.

In conclusion, it is very important that the impact of self-identified or genomic-determined African ancestry on the COAD–READ tumor immune microenvironment be evaluated while controlling for other potentially confounding co-variables. Potential links between African ancestry (self-identified or genomic) and the COAD–READ tumor immune microenvironment need to be confirmed by further assembly of recently archived COAD–READ FFPE tumor and non-tumor samples linked to rigorously curated clinical metadata and generation of linked multimodal data including both next-generation sequencing and digital imaging.

## Data Availability

The datasets presented in this study can be found in online repositories. The names of the repository/repositories and accession number(s) can be found below: https://www.ncbi.nlm.nih.gov/gap/, phs004622.v1.
