# Supplementary material for: Self-identified African ancestry and pathomics-derived tumor-infiltrating lymphocyte scores in colon and rectal adenocarcinoma whole-slide images: an exploratory multicohort whole-slide image study
Source: Front Oncol. 2026 Aug 10;16:1855135. doi: 10.3389/fonc.2026.1855135 (PMC13500275; doi:10.3389/fonc.2026.1855135)

***Supplementary Materials.***

## Supplementary Tables

**Supplementary Table S1. Descriptive table of TIL% by cohort (US vs. TCGA vs. Nigerian)**

| Cohort | N | Mean | SD | Min | Median | Max | IQR | P-value* |
|---|---|---|---|---|---|---|---|---|
| US | 97 | 19.22 | 11.33 | 2.99 | 17.40 | 56.70 | 14.85 | 0.0006 |
| TCGA | 242 | 14.62 | 8.68 | 1.95 | 13.24 | 52.53 | 11.21 | |
| Nigerian | 48 | 19.15 | 12.80 | 0.54 | 16.41 | 56.04 | 12.34 | |

*: P-value was based on Kruskal-Wallis test.

**Supplementary Table S2.Descriptive table of TIL% by combined cohort (US and TCGA) African ancestry vs. Nigerian African ancestry.**

| Cohort | N | Mean | SD | Min | Median | Max | IQR | P-value* |
|---|---|---|---|---|---|---|---|---|
| US and TCGA African | 82 | 17.98 | 11.11 | 2.03 | 15.73 | 55.81 | 14.51 | 0.5643 |
| Nigerian African | 48 | 19.15 | 12.80 | 0.54 | 16.41 | 56.04 | 12.34 | |

*: P-value was based on Wilcoxon rank sum test.

**Supplementary Table S3. Estimated differences and 95% CI in BOX-COX transformation of TIL% for ancestry, MMR/MSI status, categorical age, tumor location, tumor stage, tumor grade based on a multiple linear regression model.**

| Effect | Level | Estimated differences (95% CI) | P-value* |
|---|---|---|---|
| Ancestry | African vs European | 0.285 (0.016, 0.555) | 0.0382 |
| MMR/MSI status | MMR-d/MSI-H vs MMR-p/MSI-L+MSS | 0.274 (-0.023, 0.571) | 0.0707 |
| Categorical Age | <50 vs 50-75 | 0.023 (-0.344, 0.390) | 0.1240 |
| | <50 vs >75 | -0.272 (-0.692, 0.148) | |
| | 50-75 vs >75 | -0.295 (-0.581, -0.009) | |
| Tumor location | Right colon vs. Left colon + Rectum | 0.109 (0.155, -0.373) | 0.4174 |
| Tumor stage | I+II vs III+IV | 0.215 (-0.028, 0.458) | 0.0822 |
| Tumor grade | G1+G2 vs G3+mucinous | 0.281 (0.003, 0.559) | 0.0476 |

*: P-value was from Type 3 analysis based on a multiple linear regression model.
Note: Box-Cox transformation was applied for TIL%.

**Supplementary Table S4. Estimated differences and 95% CI in BOX-COX transformation of TIL% for ancestry*MMR/MSI status, categorical age, tumor location, tumor stage, tumor grade based on a multiple linear regression model.**

| Effect | Level | Estimated differences (95% CI) | P-value* |
|---|---|---|---|
| African vs European | MMR-d/MSI-H | 0.379 (-0.116, 0.875) | 0.6561 |
| | MMR-p/MSI-L+MSS | 0.247 (-0.072, 0.566) | |
| Categorical Age | <50 vs 50-75 | 0.027 (-0.341, 0.395) | 0.1297 |
| | <50 vs >75 | -0.266 (-0.687, 0.155) | |
| | 50-75 vs >75 | -0.293 (-0.579, -0.007) | |
| Tumor location | Right colon vs. Left + Rectum | 0.114 (-0.151,0.379) | 0.3985 |
| Tumor stage | I+II vs III+IV | 0.221 (-0.024, 0.465) | 0.0766 |
| Tumor grade | G1+G2 vs G3+mucinous | 0.279 (0.001, 0.558) | 0.0495 |

*: P-value was from Type 3 analysis based on a multiple linear regression model.
Note: Box-Cox transformation was applied for TIL%.

## Supplementary Figures

**Supplementary Figure S5. Q-Q plots.** (A) plot corresponding to linear regression model results shown in **Table 2A**. (B) plot corresponding to linear regression model results shown in **Table 3A**.

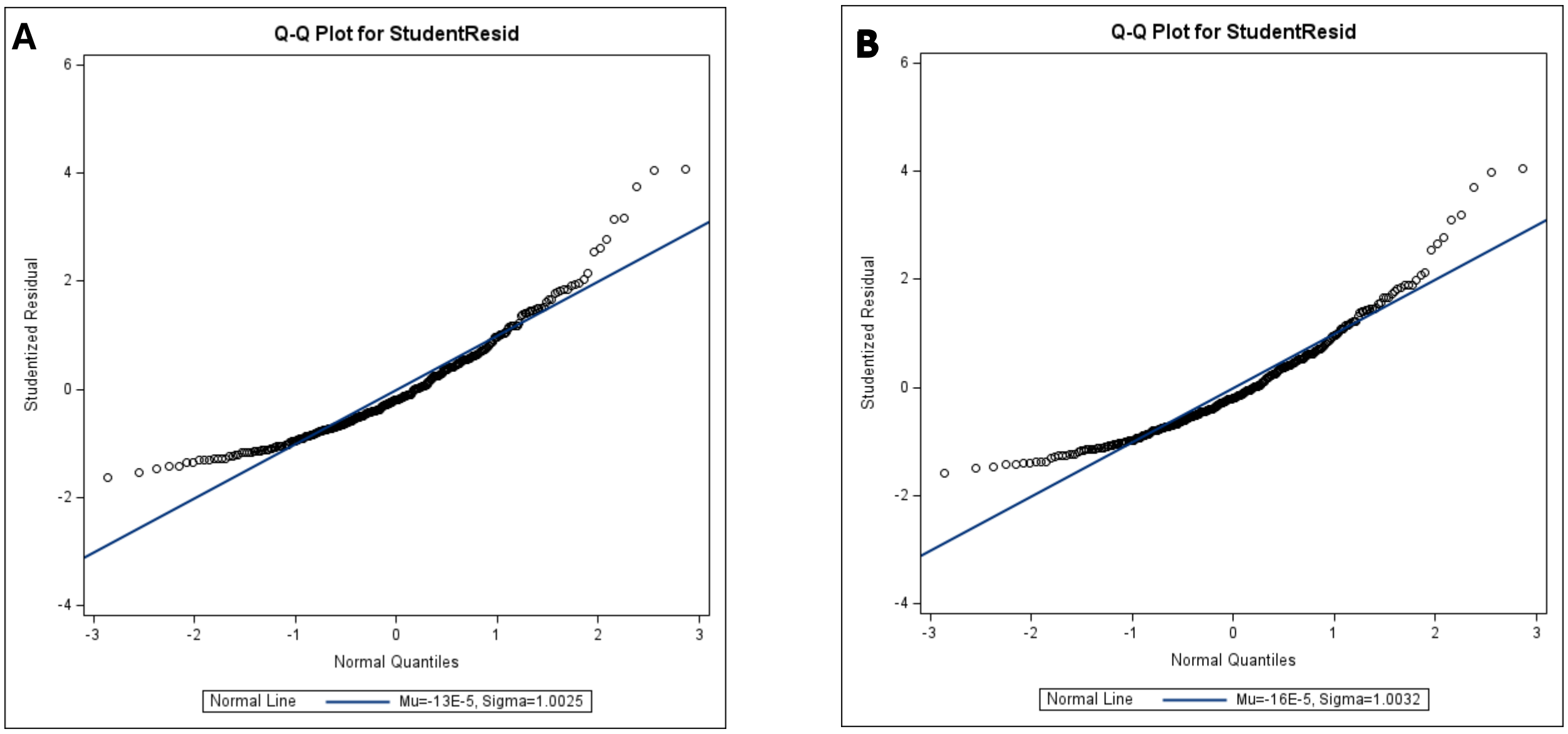


**Supplementary Figure S6. Residual vs. Predicted Value.** (A) plot corresponding to linear regression model results shown in **Table 2A**. (B) plot corresponding to linear regression model results shown in **Table 3A**.

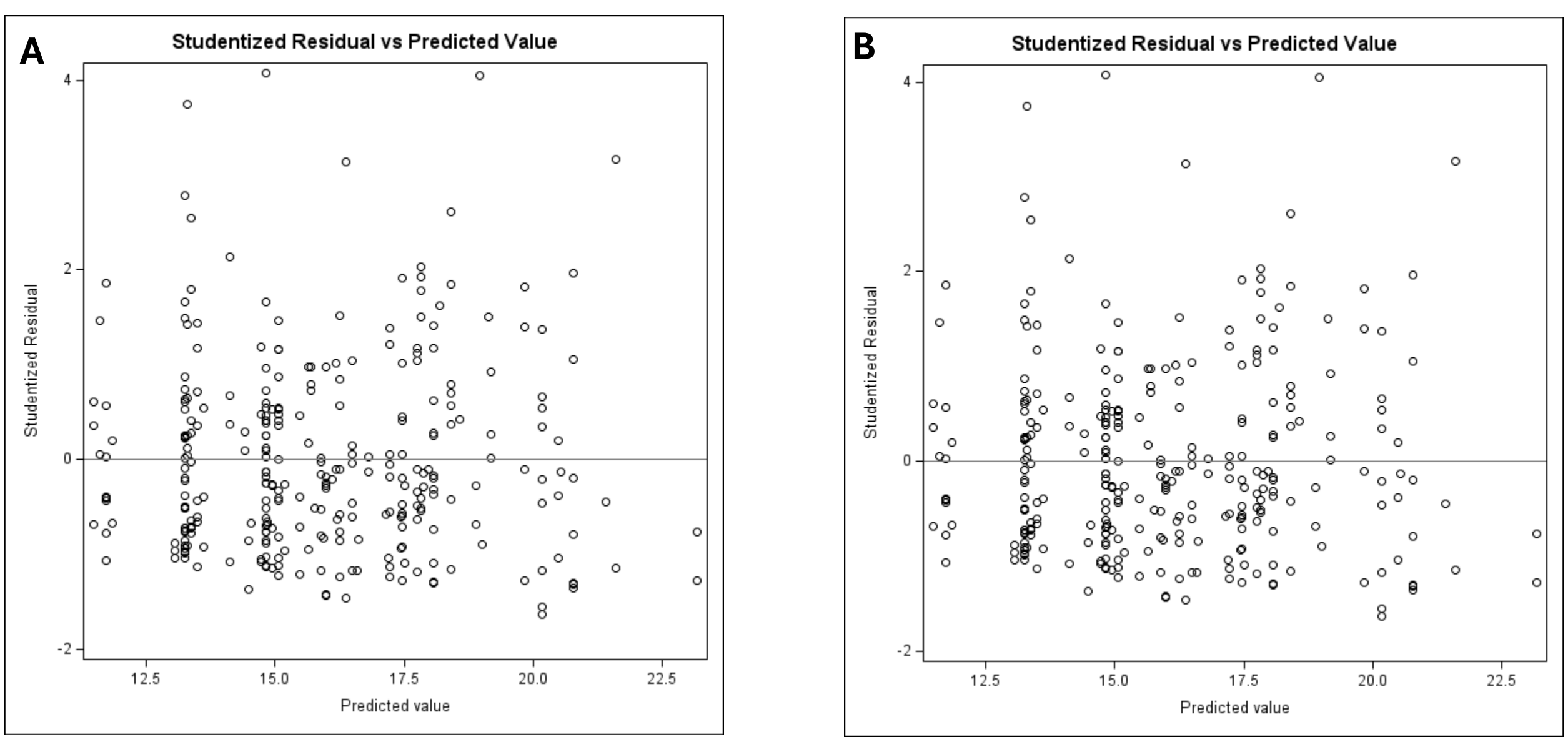

Supplement: Supplementary file 1 [file DataSheet1.pdf]
